# Squamous cell carcinoma arising from an epidermal cyst: A case series of 5 cases and literature review

**DOI:** 10.1016/j.radcr.2026.06.127

**Published:** 2026-07-22

**Authors:** Takuma Usuzaki, Sota Oguro, Shin Hitachi, Yohei Morishita, Shinichirou Yoshida, Jun Iwatsu, Munenori Watanuki, Mika Watanabe, Hiromitsu Tannai, Hiroki Kamada, Hideo Morioka, Akira Hashimoto, Kei Takase

**Affiliations:** a Department of Diagnostic Radiology, Tohoku University Hospital, Miyagi, 980-8574, Japan; b Department of Diagnostic Radiology, Japanese Red Cross Ishinomaki Hospital, Miyagi, 986-8522, Japan; c Department of Radiology, Takeda General Hospital, Fukushima, 965-8585, Japan; d Department of Orthopaedic Surgery, Tohoku University School of Medicine, Miyagi, 980-8574, Japan; e Department of Orthopaedic Surgery, JR Sendai Hospital, Miyagi, 980-8508, Japan; f Department of Pathology, Tohoku Kosai Hospital, Miyagi, 980-0803, Japan; g Department of Orthopaedic Surgery, NHO Tokyo Medical Center, Tokyo, 152-8902, Japan; h Department of Dermatology, Tohoku University Hospital, Miyagi, 980-8574, Japan

**Keywords:** Computed tomography, Epidermal cyst, Squamous cell carcinoma, Magnetic resonance imaging, Malignant transformation

## Abstract

Epidermal cysts are among the most common benign subcutaneous tumors and can undergo malignant transformation at a rate of 0.1%-2.2%. However, there are limited studies that summarize the radiological characteristics of malignant lesions arising from epidermal cysts. This study aims to report 5 patients diagnosed with malignant transformation of epidermal cysts, emphasizing the importance of radiological assessment in distinguishing benign from malignant lesions. The five patients were in their 50s to 80s, and all patients were male. The duration from the time a patient recognized a lesion to diagnosis ranged 7 and 60 years. One patient had a history of injury 18 years ago. The longest diameter of the lesion ranged from 7 to 25 cm. The lesions tended to have cystic lesions with nodules on the wall on the T2-weighted image, isointensity to muscle on the T1-weighted image, the enhanced wall and nodules on the contrast-enhanced T1-weighted image, and restricted diffusion. There were 2 important radiological findings that are associated with malignancy: the large size of the cyst and a well-enhanced nodule on the cyst wall. Additionally, the long-standing presence of the lesion may support making a diagnosis of malignant transformation. When malignancy is suspected in an epidermal cyst, radiological assessment and needle biopsy play essential roles in avoiding unplanned excision and guiding appropriate surgical management.

## Introduction

An epidermal cyst is a benign cyst that arises from the epidermis or hair follicle infundibulum and is encapsulated within a thin layer of epidermis-like epithelium [1]. The wall of the epidermal cyst is lined with stratified squamous epithelium, therefore, peeling of keratin layers accumulate inside the cyst [1,2]. Epidermal cysts tend to grow slowly during their natural course. It is commonly asymptomatic but may become symptomatic due to infection or damage to the surrounding anatomical structures [2,3]. Infected epidermal cysts cause fever and pain. Approximately 0.1%-2.2% of epidermal cysts undergo malignant transformation to squamous cell carcinoma or basal cell carcinoma [[2], [3], [4], [5]]. Previous studies have estimated that squamous and basal cell carcinoma accounts for 90% and 10% of these malignant lesions, respectively [4,5]. Although an epidermal cyst can be resected via marginal excision, it should be resected with a wide margin if the lesion contains a malignant part. Surgical treatment with appropriate margin leads to good prognosis [6]. Thus, radiological assessment of benign or malignant tumors plays an essential role in determining the surgical excision strategy and following treatment [2]. Several imaging techniques, such as ultrasound [7], radiography [8], computed tomography (CT) [9,10], and magnetic resonance imaging (MRI) [11,12] have been used to diagnose epidermal cysts. A previous study reported that irregular enhancement of the epidermal cyst wall on MRI indicated malignant transformation [11,13]. However, few studies have discussed the malignant transformation of epidermal cysts in conjunction with radiological assessments. In this study, we present 5 cases of malignant transformation of epidermal cysts and their associated imaging findings mainly focusing on CT and MRI.

## Collection of cases

Our Institutional Review Board approved this study (protocol identification number: 35864). Written informed consent was obtained from the patients’ next of kin for the three cases treated at our institution. For the remaining two cases, the requirement for informed consent was waived because the patients could not be contacted owing to outdated contact information. All 5 patients had undergone surgical resection and were histopathologically diagnosed with squamous cell carcinoma arising from epidermal cysts. In this study, data on demographic characteristics such as age, sex, clinical symptoms, tumor dimensions, and tumor sites were documented. Furthermore, radiological images of all instances were examined. This case report was written following the case report (CARE) guidelines [14].

## Case series

Table 1 shows the characteristics of the 5 patients, including the location of the lesion, medical history, and imaging findings. For case 1, 2, and 4, the lowest apparent diffusion coefficient (ADC) value was obtained at solid part.

**Table 1:** Characteristics of the patients and image findings.

Case	Age at diagnosis (y)	Sex	Location of lesion	Duration from recognition to diagnosis (y)	Histopathological diagnosis	Past injury^a^	The longest diameter (cm)	CT	T2WI	T1WI	CE-T1WI	ADC, b (10^−3^ mm^2^/s)
1	60s	Male	Left inguinal to buttock/ subcutaneous	7	Squamous cell carcinoma	None	11	-	Cystic lesion with nodules on the wall	Isointensity to muscle	Cystic walls and solid components both enhance with contrast.	1.1
2	60s	Male	Right buttock/ subcutaneous	10	Squamous cell carcinoma	None	13	Cystic lesion containing a nodular component	Cystic lesion with nodules on the wall	Isointensity to muscle	Cystic walls and solid components both enhance with contrast.	1.3
3	50s	Male	Posterior left thigh/ intermuscular	Unknown	Squamous cell carcinoma	18 y ago	7	-	Cystic lesion with a solid part on the wall	Isointensity to muscle	Cystic walls and solid components both enhance with contrast.	-
4	80s	Male	Left shoulder/ subcutaneous	60	Squamous cell carcinoma	None	25	-	Cystic lesion with a solid part on the wall	Isointensity to muscle	Cystic walls and solid components both enhance with contrast.	1.1
5	60s	Male	Left buttock/ subcutaneous	>10	Squamous cell carcinoma	None	13	Cystic lesion containing a nodular component	-	-	-	-

Abbreviations: CE-CT, contrast-enhanced computed tomography; CE-T1WI, contrast-enhanced T1-weighted image; DWI/ADC: diffusion-weighted image/apparent diffusion coefficient; T1WI, T1-weighted image; T2WI, T2-weighted image.

a A past injury implies an epidermal inclusion cyst.

b The DWI/ADC reported in Table 1 was the lowest value in the enhanced region of the CE-T1WI.

### Case 1

#### Patient history

A man in his 60s had swelling in his left inguinal region up to the left breech for 7 years since noticing the mass for the first time, which had grown rapidly for the past 3 months. His medical history included appendicitis, a subarachnoid hemorrhage, and hypertension. Marginal excision under general anesthesia was performed at a previous medical facility. Histopathological examination confirmed that the mass was a squamous cell carcinoma, and the surgical margin was positive. Therefore, the patient was referred to our hospital, and additional wide resection were performed.

#### Imaging findings

**Fig. 1:**
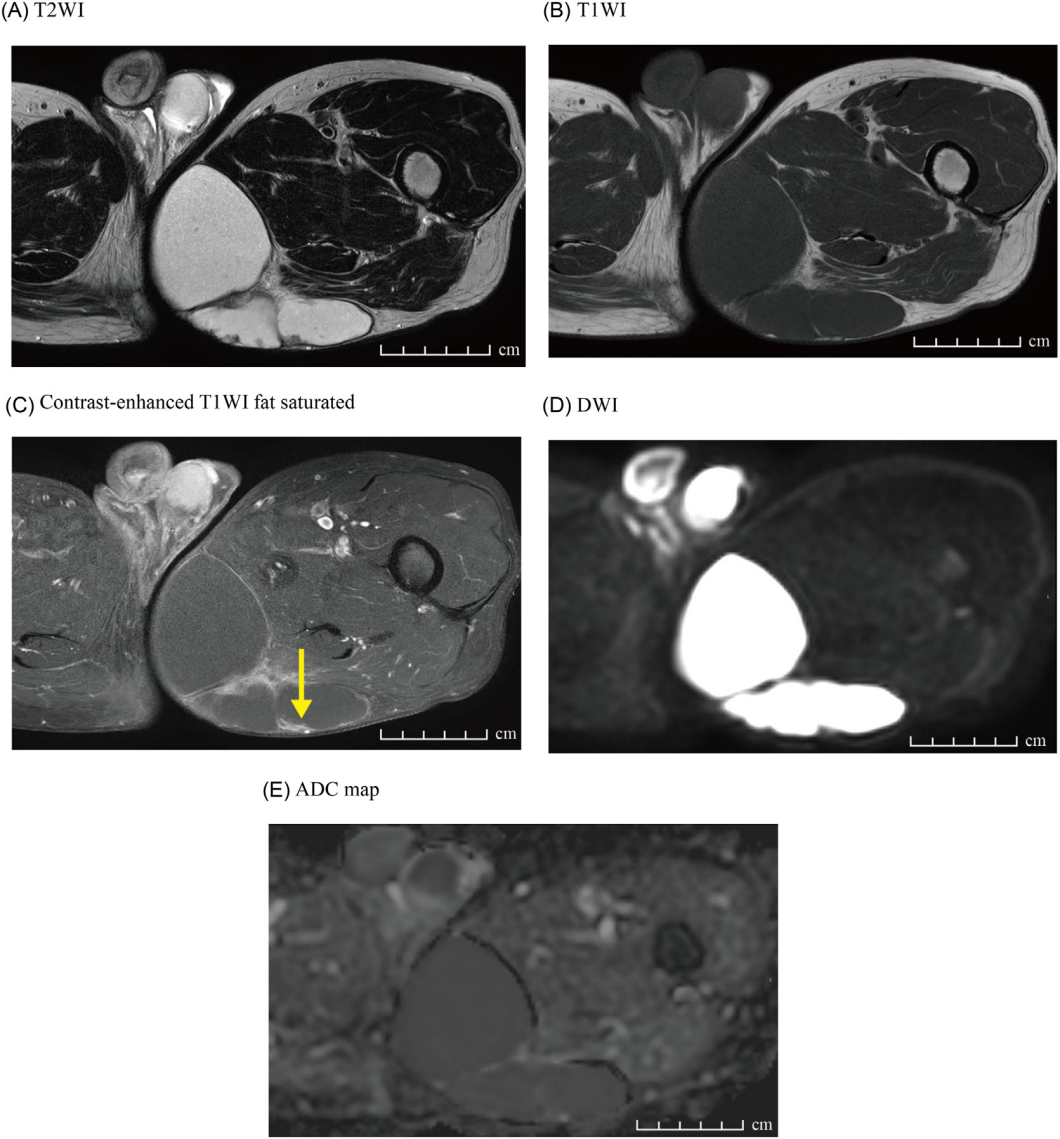
Case 1, a male in his 60s. Magnetic resonance imaging (MRI) is shown: (A) T2-weighted image (T2WI), (B) T1-weighted image (T1WI), (C) contrast-enhanced T1WI fat-saturated (a nodule is indicated by yellow arrow), (D) diffusion-weighted image (DWI), and (E) apparent diffusion coefficient (ADC) map.

Preoperative magnetic resonance imaging (MRI) from the previous hospital demonstrated a multilocular cystic tumor measuring 11 × 7 cm from the left inguinal region to the left breech. Most lesions showed a high signal intensity on T2-weighted images (T2WI), with low signal intensity nodular lesions observed along the wall (Fig. 1A). The nodular lesions appeared as iso-intensity signal to muscle on T1-weighted images (T1WI) and demonstrated contrast enhancement (Figs. 1B and C). On diffusion-weighted imaging (DWI) and ADC map, the entire lesion exhibited a high and low signal intensity, respectively (Figs. 1D and E).

#### Pathological findings

The pathological findings of the resected tumor reveal squamous cell carcinoma with marked keratinization is observed. The tumor appears to extend along the cyst wall and is surrounded by fibrous tissue. Tumor nests are also found within the surrounding fibrous tissue in some areas, suggesting invasion.

### Case 2

#### Patient history

A man in his 60s had a mass in his right breech that had gradually grown over 10 years. Owing to pain in the same area, the patient consulted a previous medical provider. He had no relevant medical history. At the previous medical facility, he had been diagnosed with a subcutaneous cystic tumor, and marginal excision was performed under general anesthesia. On histopathological examination, the lesion was diagnosed as a squamous cell carcinoma arising from an epidermal cyst, and the surgical margins were found positive. Therefore, the patient was referred to our hospital, and additional wide resection were performed.

#### Imaging findings

**Fig. 2:**
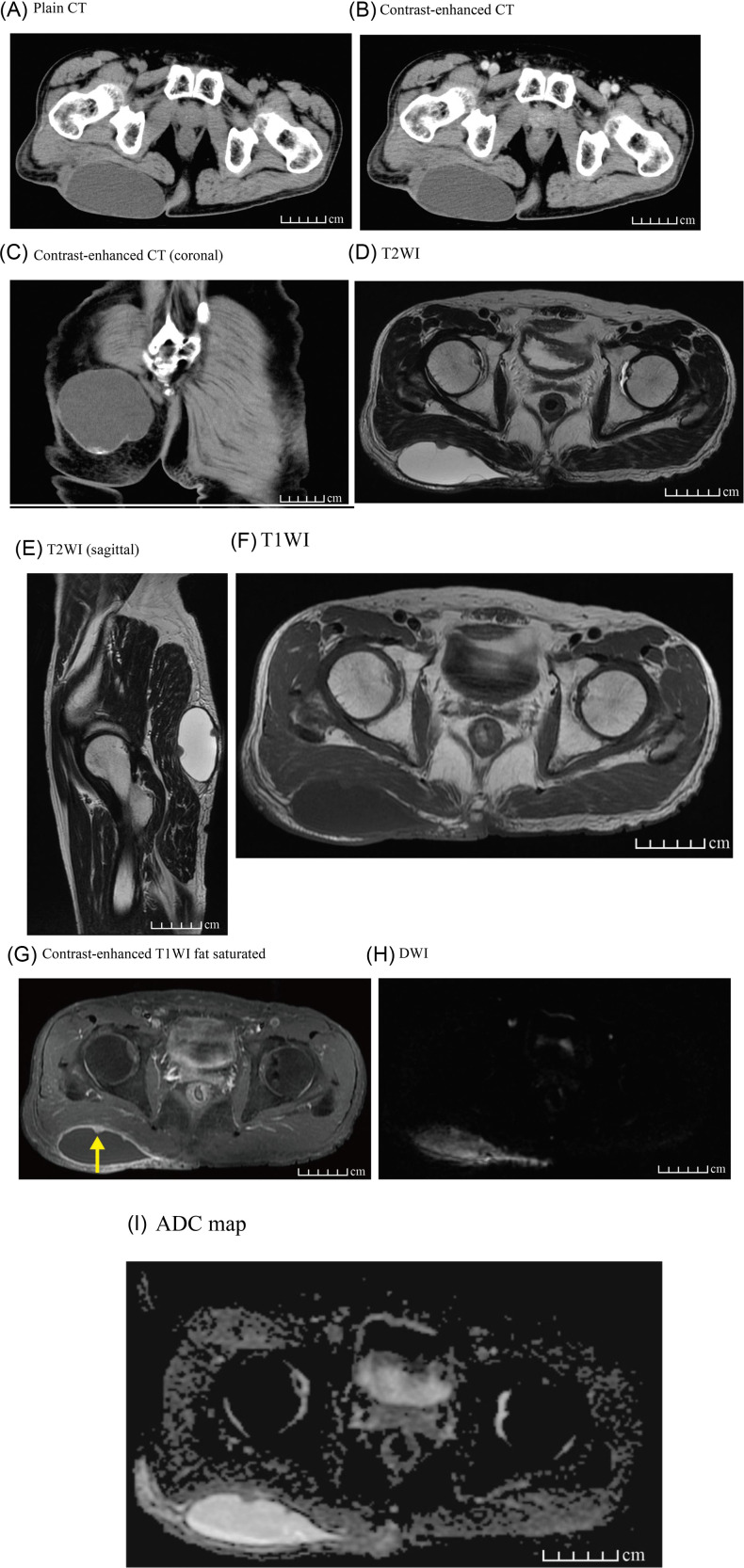
Case 2, a male in his 60s. Computed tomography images are shown in (A) plain CT, (B) contrast-enhanced CT, and (C) contrast-enhanced CT on the coronal plane. Magnetic resonance imaging (MRI) is presented in (D) T2-weighted image (T2WI), (E) T2WI on the sagittal plane, (F) T1-weighted image (T1WI), (G) contrast-enhanced T1WI fat-saturated (a nodule is indicated by yellow arrow), (H) diffusion-weighted image (DWI) and (I) apparent diffusion coefficient (ADC) map.

Preoperative contrast-enhanced computed tomography revealed a cystic tumor measuring 13 × 6 cm at his right breech. A nodular lesion was observed on the cyst wall (Figs. 2A-C). MRI at the time of recurrence revealed a cystic tumor measuring 10×4 cm at his right breech. A cystic tumor was observed predominantly within the subcutaneous adipose tissue with multiple nodular lesions visible on the wall and showing low signal intensity on T2WI (Figs. 2D and E). Most lesions appeared as iso-intensity signals to muscle on T1WI (Fig. 2F), and the nodular lesions exhibited substantial contrast enhancement (Fig. 2G). The nodular lesions exhibited restricted diffusion (Fig. 2H and I).

#### Pathological findings

The pathological findings of the resected tumor reveal a well-differentiated squamous cell carcinoma with marked keratinization, extending from subcutaneous tissue to skeletal muscle. The tumor is near the deep margin and may represent residual disease from a previous excision, as it is found adjacent to scar tissue and surgical sutures.

### Case 3

#### Patient history

A man in his 50s consulted a physician for a mass in the posterior left thigh that had gradually grown. He had an injury to the posterior left thigh caused by a weed cutter 18 years previously. His medical history included infiltrating urothelial carcinoma and benign prostatic hyperplasia. At another hospital, the patient was diagnosed with a cystic tumor, and marginal excision was performed under general anesthesia. On histopathological examination, the lesion was diagnosed as a squamous cell carcinoma arising from an epidermal inclusion cyst, and the surgical margins were positive.

#### Imaging findings

**Fig. 3:**
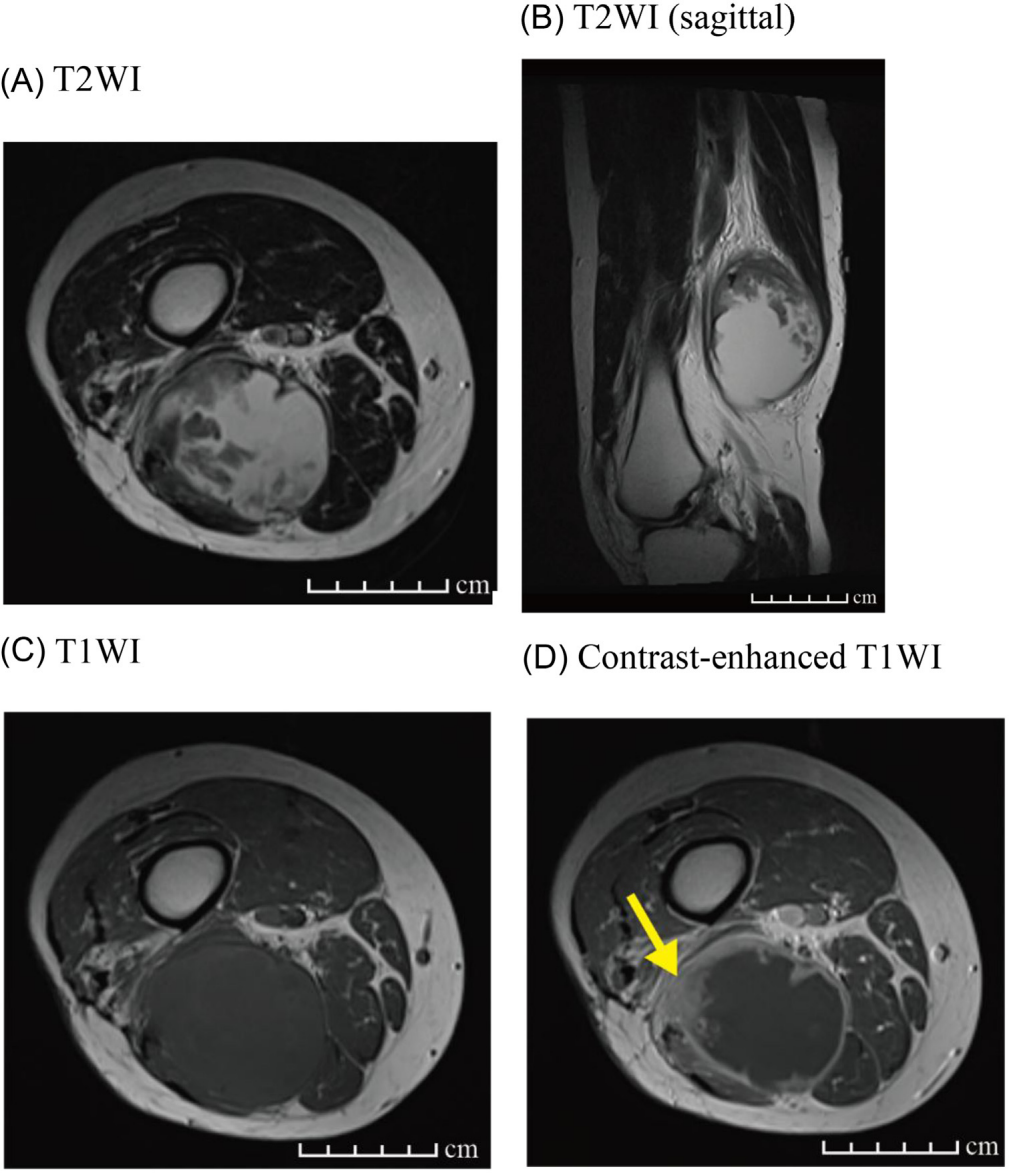
Case 3, a male in his 50s. Magnetic resonance imaging (MRI) is shown; (A) T2-weighted image (T2WI), (B) T2WI on sagittal plane, (C) T1-weighted image (T1WI), and (D) contrast-enhanced T1-weighted image (an enhancing mural nodule is indicated by the yellow arrow).

Preoperative MRI performed at another hospital revealed a cystic tumor measuring 7 × 6 cm at his posterior left thigh. The tumor showed heterogeneous intensity on T2WI (Figs. 3A and B) and iso-signal intensity to muscle on T1WI (Fig. 3C). On contrast-enhanced T1WI, the wall of the cyst and nodular lesion on the wall were well-enhanced (Fig. 3D). He was referred to our hospital, where additional wide resection and radiation therapy were performed. The resected specimen revealed a cystic lesion measuring approximately 7 cm.

#### Pathological findings

Histologically, the lesion exhibited squamous cell carcinoma with keratinization, chromatin clumping, and eosinophilic cytoplasm proliferating within a cystic structure, with minimal invasion into the underlying skeletal muscle. Inflammatory cell infiltration was also noted in the surrounding tissue.

### Case 4

#### Patient history

A man in his 80s presented with a tumor on his left shoulder. The size of the tumor was approximately 5 cm and had not changed for 60 years; however, it rapidly expanded in the past 1 year.

#### Imagimg findings

**Fig. 4:**
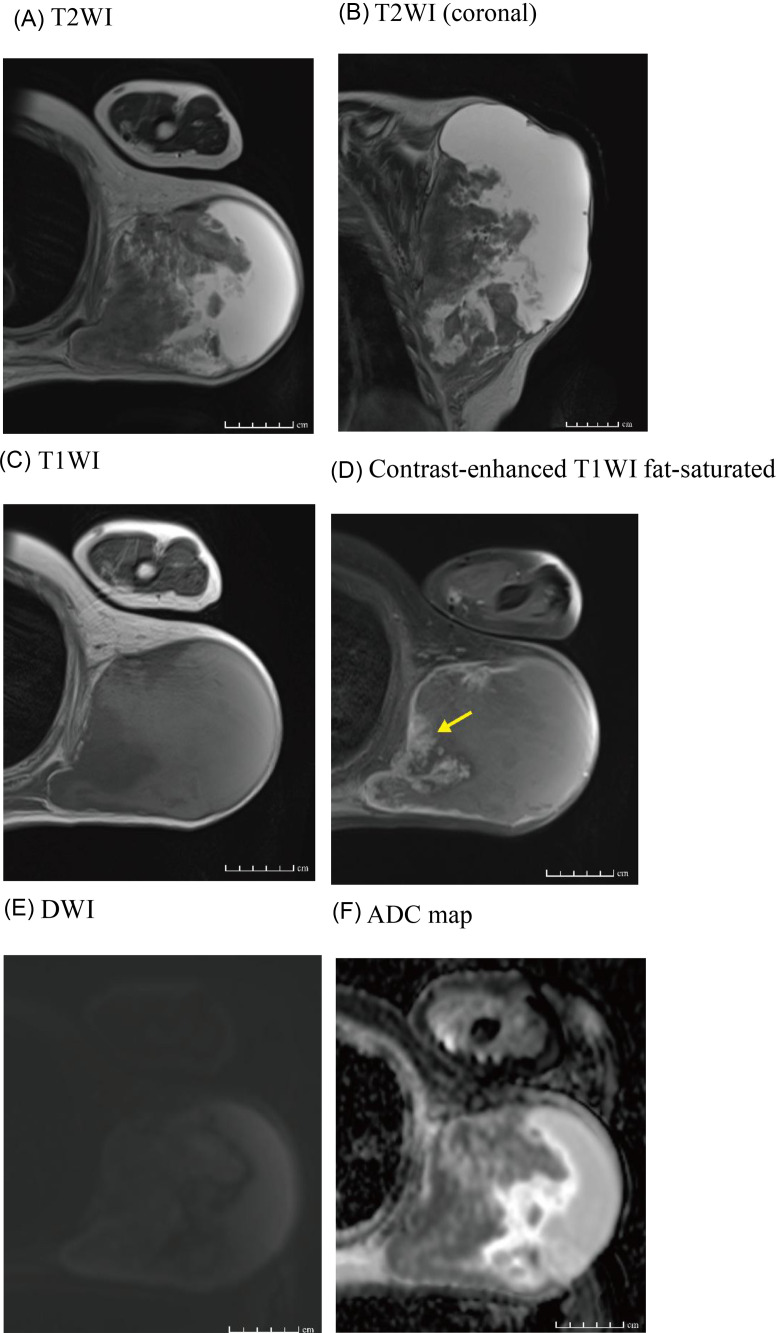
Case 4, a male in his 80s. Magnetic resonance imaging (MRI) is shown; (A) T2-weighted image (T2WI), (B) T2WI on coronal plane, (C) T1-weighted image (T1WI), (D) contrast-enhanced fat-saturated T1-weighted image (an enhancing mural component corresponding to the area of restricted diffusion is indicated by the yellow arrow), (E) diffusion-weighted image, and (F) apparent diffusion coefficient map.

Preoperative MRI at a previous hospital revealed a subcutaneous tumor measuring 25 × 12 cm on his left shoulder. The tumor showed a heterogeneous signal with a dendritic low-signal area on the tumor wall on T2WI (Figs. 4A and B). The tumor edges were smooth. On T1WI, the tumor showed iso-intensity to muscle (Fig. 4C), and contrast-enhanced T1WI fat saturation showed partial enhancement at the dendritic low-signal area (Fig. 4D). DWI exhibited entirely low signal and low area in ADC map was observed corresponding the contrast enhanced area (Figs. 4E and F).

#### Pathological findings

Needle biopsy revealed squamous cell carcinoma arising from the epidermis. Based on this diagnosis, the tumor was resected. The surgical margins were negative, indicating complete removal of the malignant tissues.

### Case 5

#### Patient history

A 60s male presented with 2 contiguous bilobed cystic tumors measuring 9 × 5 cm and 7 × 7 cm on his left buttock. These tumors had been monitored for over a decade and showed no significant changes. The patient had no significant medical history.

#### Imaging findings

Prior to surgery, a computed tomography (CT) scan was performed, which identified a well-demarcated bilocular mass measuring 13×4 cm in the left buttock area with a density of 26 HU, suggestive of an epidermal cyst. Thirteen years after the onset, owing to a recent increase in tumor size and discomfort, the patient underwent surgical intervention. Marginal excision of the tumors was performed under general anesthesia.

#### Pathological findings

Histopathological examination revealed an epidermal cyst with a well-differentiated squamous cell carcinoma on the wall. The surgical margins were negative, indicating complete removal of the malignant tissues.

## Discussion

Although the terms epidermal cyst and epidermoid cyst are often used interchangeably in medical texts and articles, epidermal cyst is the correct term [1,15]. Hoang et al. described the difference between these terms in their review [1]. In the present study, we reported 5 cases of malignant lesions arising from epidermal cysts and the radiological assessment of each patient through presenting images. To the best of our knowledge, approximately 70 cases (including our cases) who had malignant lesion arising from epidermal cyst were reported [6,16]. In the largest case series, the average at diagnosis was 57.3 years, the male/female ratio was 7:2, and the average latency period of 15.4 years [6]. A novelty of the present study is reporting radiological assessment mainly focusing on CT and MRI. Four of the 5 patients were suspected to have benign epidermal cysts. However, histopathological examination revealed malignant transformation of the epidermal cysts in all 5 cases. For 3 out of 5 cases, the initial surgery was an “unplanned resection” without needle biopsy. When a lesion is malignant, unplanned resection leads to additional wide resection because of the contamination of the surrounding normal tissue by malignant cells during surgery. Contamination may cause local iatrogenic recurrence and metastases. Moreover, additional wide resection sometimes result in the deterioration of the quality of life of patients. Malignancies should be carefully assessed to provide the best treatment for patients. In terms of assessment, we believe that not only histopathological assessments by biopsy but also radiological assessments play an essential role. The imaging findings presented in this study indicate that 2 key imaging findings may be able to distinguish the malignant transformation of epidermal cysts from benign epidermal cysts: lesion size and well-enhanced nodules on the cyst wall.

The first key finding of malignant lesions arising from epidermal cysts is the size of the lesion. The size of epidermal cysts varies. A size of squamous cell carcinoma arising from epidermal cyst was reported as 1.5-14.3 cm in the longest diameter [6,16,17]. Lee et al. analyzed 17 benign epidermal inclusion cysts using ultrasound and reported that the mean longest diameter was 3.1 cm ranging from 1 to 6 cm [18]. Shibata et al. collected 5 patients with benign epidermal inclusion cysts and reported that the size of the lesions on MRI ranged from 2 to 10 cm, with an average of 5.2 cm [11]. The lesions reported in the present study were relatively larger than those reported previously. Malignancy is associated with the ability to proliferate, and malignant lesions arising from epidermal cysts tend to be larger than benign lesion [16]. In addition to size, the increase in size may be a main symptom associated with malignancy [6]. The lesion size is a reproducible index that can be easily examined. Biopsy to rule out malignancy should be considered when epidermal cysts are larger than the typical size reported previously.

The second key imaging finding of a malignant lesion arising from an epidermal cyst is a well-enhanced nodule on the cyst wall on MRI. Kawauchi et al. reported that irregular enhancement of the marginal region of an epidermal cyst on MRI is a key finding of malignant transformation [11,13]. In CT and MRI, malignant lesions reported in the present study had well enhanced nodule on the cyst wall. For benign epidermal cysts, CT demonstrates a well-encapsulated mass of heterogeneous density, representing a mixture of fat and keratin [10]. In addition, an unruptured benign epidermal cyst was identified as a noninfiltrating, fluid-dense mass with a thin sclerotic wall. The wall can be enhanced using contrast-enhanced materials [9]. These properties were confirmed in 5 patients in this study. In contrast, the MRI of benign unruptured epidermal cysts reported in previous studies shows a well-defined mass that represents a high background signal with an internal low signal on T2WI and T1WI [12]. Compared to muscular signals on MRI, epidermal cysts often show hypo- or iso-intensisty signals on T1WI. T1WI imaging reveal slightly hyperintense foci. Contrast-enhanced T1WI reveals a mass with central nonenhancement and peripheral thin-rim enhancement [9,19]. Diffusion-weighted imaging (DWI) showed that the signal intensities of the subcutaneous epidermal cysts were high, whereas the apparent diffusion coefficient (ADC) values were low. The ADC values observed in our cases were 1.1, 1.3, and 1.1 × 10^−3^ mm^2^/s. Previous studies reported the mean ADC values for abscesses and malignant soft tissue tumors were 0.877 (95% confidence interval 0.697-1.17) and 0.449 (standard deviation 0.27) × 10^−3^ mm/s, respectively [20,21]. Based on the results, the ADC value of a malignant lesion arising from an epidermal cyst may be similar to or higher than that of abscesses and higher than that of malignant soft tissue tumors. Interestingly, the apparent diffusion coefficient values of subcutaneous epidermal cysts were significantly lower than those of intracranial epidermal cysts. However, similar to the CT findings, the malignant lesion arising from the epidermal cysts had a well-enhanced nodule on the cyst wall, as observed on contrast-enhanced T1WI. In all patients for whom MRI data were available, well-enhanced nodules were observed on contrast-enhanced T1WI. Malignancy should be suspected when a well-enhanced nodule is detected within the cyst walls.

Considering the above 2 key findings and typical imaging findings of epidermal cysts. We can differentiate several diseases. Notably, we can differentiate soft-tissue sarcoma and infected cyst/abscess from malignant transformation of epidermal cysts based on 2 key findings and ADC values [20,21]. A chronic expanding hematoma shows T1WI hyperintensity relative to the muscle signal [22,23]. Myxoid tumors show variable heterogeneous contrast enhancement due to the presence of abundant vascularity within the myxoid matrix [24,25]. Nodular fasciitis represents fast growth; however, it is typically small (<2 cm) [26]. Proliferating trichilemmal tumors often occur on the scalp, predominantly in middle-aged women [27]. This shows T1WI isointense signal and T2WI hyperintense. Contrast-enhanced MR shows significant enhancement with portions remaining unenhanced [28]. A key finding of the ruptured epidermal cyst thick and irregular rim [29].

Ultrasonography is another modality to evaluate the property of a lesion [6]. On ultrasonography, the epidermal cyst usually presents as a well-circumscribed, hypoechoic mass with no vascularity located at or deep within the skin. Kim et al. [6] reviewed 9 cases with malignant lesions arising from epidermal cyst. They reported that 2 out of 3 cases evaluated by ultrasonography presented a typical feature of epidermal cyst. Although ultrasonography has limited roles in determining malignancy, it can be useful for biopsy.

It is controversial whether past injury affect the location of epidermal cysts. The case 3 had the malignant lesion located at inter-muscles on MRI. This case implied that stratified squamous epithelium was left at inter-muscles after injury and resulted in malignant transformation. This hypothesis can be supported by a case reported by Kubota et al. [30]. They reported an epidermal cyst in the rectus abdominis muscle after surgery. However, the detail of injury is unknown, and we reported only 1 case of malignant lesion arising from the inclusion cyst. It is necessary to accumulate more cases to clarify the relation between location of malignant lesion and injury.

The pathological differentiation of malignant transformation of epidermal cysts from that of trichilemmal cysts could be challenging because of the histological similarity and same hair follicular origin of both cysts. There are several points to consider when diagnosing malignant transformation of epidermal cysts. First, it is important that the predisposing epidermal cyst has been clinically observed over a long duration. Second, a benign squamous epithelium can be observed in the same capsule-like structure as a squamous cell carcinoma or basal cell carcinoma, confirming a predisposing benign cystic lesion. All 5 cases in this study had been diagnosed as an epidermal cyst over a long period, averaging 13 years, with the longest duration being 60 years. Tanaka et al. concluded in their case report that histological examination should be considered for longstanding epidermal cysts because of the potential of malignant transformation [4]. Furthermore, squamous cell carcinoma was pathologically identified during excisional surgery, leading to the diagnosis of malignant transformation of the epidermal cyst.

## Conclusion

In conclusion, we report 5 patients with malignant lesions arising from epidermal cysts. Two key radiological findings found to be associated with malignancy were large size of the cyst and a well-enhanced nodule on the cyst wall. When these 2 findings are observed, a radiologist should recommend a biopsy. Additionally, the long-standing presence of the lesion should be considered an important feature that should raise suspicion for malignant transformation of an epidermal cyst. When malignancy is suspected in an epidermal cyst, radiological assessment and needle biopsy play essential roles in avoiding unplanned excision and guiding appropriate surgical management.

## Author contribution

Data collection was performed by Sota Oguro, Shin Hitachi, Shinichirou Yoshida, Jun Iwatsu, Munenori Watanuki, Hideo Morioka, Akira Hashimoto, Yohei Morishita, and Takuma Usuzaki. Radiological interpretations were performed by Sota Oguro, Shin Hitachi, Yohei Morishita, Hiromitsu Tannai, Hiroki Kamada, Kei Takase, and Takuma Usuzaki. Pathological interpretation was performed by Mika Watanabe. Takuma Usuzaki and Sota Oguro wrote the first draft of the manuscript, and all authors commented on previous versions. All authors have read and approved the final manuscript. Takuma Usuzaki and Sota Oguro equally contributed to this manuscript as the first authors.

## Data availability statement

Due to the nature of this research, we cannot share publicly the data.

## Ethical approval

Our Institutional Review Board approved this study (protocol identification number: 35864).

## Patient consent

Written informed consents for 3 cases treated in our hospital were obtained from the next of kin of the patients. For the remaining 2 cases treated at other hospitals, IRB approval was obtained with the waiver of informed consent because the contact information on the 2 cases was too old to collect.
